# Tumour necrosis factor and PI3-kinase control oestrogen receptor alpha protein level and its transrepression function

**DOI:** 10.1038/sj.bjc.6601541

**Published:** 2004-02-17

**Authors:** P Bhat-Nakshatri, R A Campbell, N M Patel, T R Newton, A J King, M S Marshall, S Ali, H Nakshatri

**Affiliations:** 1 Walther Oncology Center, Indiana University School of Medicine, Indianapolis, IN 46202, USA; 2 Department of Surgery, Indiana University School of Medicine, Indianapolis, IN 46202, USA; 3 Department of Biochemistry and Molecular Biology, Indiana University School of Medicine, Indianapolis, IN 46202, USA; 4 Department of Cancer Medicine, Imperial College School of Medicine, Hammersmith Hospital, London W12 0NN, UK; 5 Walther Cancer Institute, Indianapolis, IN 46208, USA

**Keywords:** oestrogen receptor, tumour necrosis factor, PI3-kinase, IL-6, transrepression

## Abstract

Oestrogen receptor alpha (ER*α*) is an oestrogen-activated transcription factor, which regulates proliferation and differentiation of mammary epithelial cells by activating or repressing gene expression. ER*α* is a critical prognostic indicator and a therapeutic target for breast cancer. Patients with tumours that express higher level of ER*α* have better prognosis than patients with tumours that are ER*α* negative or express lower level of ER*α*. Better prognosis in ER*α*-positive patients is believed to be due to repression of proinvasive gene expression by ER*α*. Oestrogen receptor alpha represses gene expression by transrepressing the activity of the transcription factors such as nuclear factor-kappaB or by inducing the expression of transcriptional suppressors such as MTA3. In this report, we show that ER*α* transrepresses the expression of the proinvasive gene interleukin 6 (IL-6) in ER*α*-negative MDA-MB-231 breast cancer cells stably overexpressing ER*α*. Using these cells as well as ER*α*-positive MCF-7 and ZR-75-1 cells, we show that tumour necrosis factor alpha (TNF*α*) and the phosphatidylinositol-3-kinase (PI3-kinase) modulate transrepression function of ER*α* by reducing its stability. From these results, we propose that TNF*α* expression or PI3-kinase activation lead to reduced levels of ER*α* protein in cancer cells and corresponding loss of transrepression function and acquisition of an invasive phenotype.

Oestrogen receptor alpha (ER*α*) expression status is of prognostic significance for breast cancer. Breast cancer patients with the highest levels of ER*α* protein have a 90% 5-year survival rate and display very few p53 mutations. Patients with lower ER*α* levels have ∼45% 5-year survival and higher p53 mutation rates. The survival rate in these patients is similar to patients with a subset of ER*α*-negative breast cancer (Sorlie *et al*, 2001). Better prognosis in ER*α*-positive breast cancer patients can partly be attributed to their response to antihormone therapy (Ali and Coombes, 2002). However, because patients with higher rather than lower ER*α* protein have better prognosis, it is likely that some of the ER*α*-regulated genes suppress invasion and metastasis of breast cancer. Consistent with this possibility, it was shown recently that ER*α*-dependent expression of metastasis associated protein 3 (MTA3) is required to prevent invasive growth of breast cancer cells (Fujita *et al*, 2003). Furthermore, a recent microarray study has shown that among ∼400 genes regulated by ER*α*/oestrogen in MCF-7 cells, majority of them (70%) are downregulated (Frasor *et al*, 2003). Some of the downregulated genes are known to be involved in invasion and homing of metastatic cancer cells (Muller *et al*, 2001).

Oestrogen receptor alpha is an oestrogen-activated transcription factor, which modulates gene expression by binding to oestrogen response elements (ERE) in the responsive promoter and through protein–protein interactions (Mangelsdorf *et al*, 1995; Di Croce *et al*, 1999). Oestrogen receptor alpha contains a central DNA binding domain (DBD), C-terminal ligand binding domain (LBD), as well as ligand-dependent activation function (AF-2) and N-terminal ligand-independent activation function (AF-1). Upon binding to oestrogen, ER*α* homodimers bind to ERE in the responsive gene promoters and activate gene expression. In addition, ER*α* homodimers activate non-ERE containing promoters by interacting with transcription factors such as SP-1 and AP-1 (Gaub *et al*, 1990; Paech *et al*, 1997; Dong *et al*, 1999). Transactivation by ER*α* involves ligand-dependent recruitment of coactivators, which serve as an intermediate between the receptor and the RNA polymerase II transcription complex (Horwitz *et al*, 1996; Glass and Rosenfeld, 2000). Although binding of oestrogen to LBD is essential for complete activation of ER*α*, phosphorylation by extracellular signal-activated kinases is thought to play a role in oestrogen-dependent and oestrogen-independent activity of ER*α* (Ali *et al*, 1993; Le Goff *et al*, 1994). Recently, a novel cell-type specific nongenomic action of ER*α* involving oestrogen-dependent association of ER*α* with phosphatidylinositol-3-kinase (PI3-kinase) leading to activation of the cell survival kinase AKT has also been reported (Simoncini *et al*, 2000). In addition, ER*α* localised in the plasma membrane has been shown to activate the MAP kinase pathway and contribute to growth regulation of breast cancer cells (Filardo *et al*, 2000; Marquez and Pietras, 2001; Razandi *et al*, 2003).

Transrepression of gene expression through protein–protein interaction is also a critical function of ER*α*. For example, inhibition of GATA-1-mediated transcription by ER*α* is responsible for suppression of erythroid differentiation by oestrogen (Blobel *et al*, 1995). The protective effect of oestrogen against sepsis is believed to be due to the suppression of proinflammatory gene expression (Schroder *et al*, 1998; Evans *et al*, 2002). Similarly, ER*α*-dependent repression of nuclear factor kappa B (NF-*κ*B) activity is important for maintaining bone density (Jilka *et al*, 1992; Pottratz *et al*, 1994; Stein and Yang, 1995). Evans *et al* (2002) have identified several NF-*κ*B-regulated genes that are repressed by ER*α*, which include antiapoptotic proteins GADD45*β*, apoptosis inhibitor 2, A20 and NF-*κ*B p105 . A recent report suggested that ER*α* at higher levels reduces cancer cell growth and angiogenesis by inhibiting the expression of vascular endothelial growth factor (Ali *et al*, 2001). Furthermore, it was reported that unliganded and liganded ER*α* reduce cancer cell migration and invasion, through a mechanism that involves protein–protein interaction (Platet *et al*, 2000). Unlike the case of the glucocorticoid receptor (GR) where transrepression function is well characterised (Nissen and Yamamoto, 2000), the mechanism of ER*α*-mediated transrepression is not completely understood. Antagonism of NF-*κ*B activity has been used as a model system to understand ER*α*-mediated transrepression. Repression of NF-*κ*B activity by ER*α* is cell type specific (Cerillo *et al*, 1998). Both the DBD and LBD of ER*α* are essential for efficient repression of NF-*κ*B activity (Stein and Yang, 1995; Valentine *et al*, 2000). It is suggested that ER*α* interacts directly with the Rel-homology domains (RHD) of the NF-*κ*B subunits, p50 and p65, thereby interfering with the transcriptional activity of DNA-bound NF-*κ*B (Stein and Yang, 1995). Apart from inhibition through direct protein–protein interaction, competition for the limiting amount of common coactivators such as SRC-1 and p300/CBP is suggested to play a role in ER*α*-dependent repression of NF-*κ*B activity, although studies with ER*α* harbouring mutations in its transactivation domain fail to support such a mechanism (Sheppard *et al*, 1999; Harnish *et al*, 2000; Valentine *et al*, 2000).

The goals of this study were to determine whether ER*α* transrepresses the expression of interleukin 6 (IL-6), a cytokine that is linked to breast cancer cell invasion and motility as well as resistance to chemotherapy (Tamm *et al*, 1991; Conze *et al*, 2001), and to identify signalling pathways that may modulate transrepression by altering the stability of ER*α*. Using the ER*α*-negative breast cancer cell line MDA-MB-231 stably overexpressing ER*α*, we show that ER*α* transrepresses tumour necrosis factor alpha (TNF*α*)-inducible expression of IL-6. We also show that TNF*α* and PI3-kinase pathway modulate transrepression by reducing the stability of ER*α*.

## MATERIALS AND METHODS

### Generation of ER*α*-overexpressing cells

MCF-7, ZR-75-1 and MDA-MB-231 cells were purchased from the American Type Culture Collection (ATCC, Manassas, VA, USA). The cDNA encoding ER*α* was cloned into the *Eco*RI site of the retroviral vector LxSN (pLxSN-ER*α*) (Miller and Rosman, 1989). Packaging of retrovirus and infection of MDA-MB-231 were performed as described previously (Newton *et al*, 1999). Briefly, AM12 cells were transfected with pLxSN or pLxSN-ER*α* expression vector and selected in media containing 600 *μ*g ml^−1^ G418. G418-resistant colonies were pooled and media supernatant with virus was used for infecting MDA-MB-231 cells. MDA-MB-231 cells were incubated with viral supernatant for 2 h in the presence of 8 *μ*g ml^−1^ polybrene. The transduced cells were grown in the presence of G418 (1 mg ml^−1^). Individual G418-resistant colonies were isolated and ER*α* expression was measured by Western blotting. The constitutively active PI3-kinase expression vector (myr-PI 3-Kp110) was purchased from Upstate Biotechnology (Charlottesville, VA, USA). Constitutively active AKT (CA-AKT) and kinase-dead AKT (KD-AKT) have been described previously (Campbell *et al*, 2001). Cells were transfected with expression vectors using Lipofectamine 2000 transfection reagent as recommended by the manufacturer and analysed for ER*α* protein levels 48 h after transfection (Invitrogen, Carlsbad, CA, USA).

### Northern blot analysis

Total RNA was prepared using the RNAeasy kit from Qiagen (Valencia, CA, USA). RNA was subjected to Northern blot analysis as previously described (Newton *et al*, 1999). Interleukin-6 cDNA was purchased from ATCC, whereas tomour necrosis factor receptor associated protein 1 (TRAF-1) cDNA has been described previously (Rothe *et al*, 1994).

### Western blot analysis

Whole-cell extracts were prepared in radioimmunoassay buffer (RIPA; 50 mM Tris pH 7.5, 0.25% sodium deoxycholate, 1% NP40, 150 mM NaCl, 1 mM EDTA, 100 *μ*M sodium orthovanadate, 1 mM sodium fluoride, 1 mM *β*-glycerophosphate, 0.5 mM PMSF, 2 *μ*g ml^−1^ each of aprotenin, leupeptin and pepstatin) and subjected to Western blot analysis as previously described (Newton *et al*, 1999). Oestrogen receptor alpha antibody raised against B-domain of ER*α* was purchased from Chemicon (MAB463; Temecula, CA, USA), whereas *β*-actin antibody was from Sigma Chemicals (St Louis, MO, USA). MG132, PD98059, LY294002, PP2 and protein kinase A (PKA) inhibitor peptide were purchased from Calbiochem (San Diego, CA, USA), whereas TNF*α* was purchased from R&D systems (Minneapolis, MN, USA). Cells were incubated with kinase inhibitors for 2 h before addition of TNF. 4-Hydroxytamoxifen was purchased from Sigma Chemicals, whereas ICI182780 was purchased from Tocris (Ellisville, MO, USA).

## RESULTS

### ER*α* reduces TNF*α*-inducible IL-6 but not TRAF-1 expression

**Figure 1:**
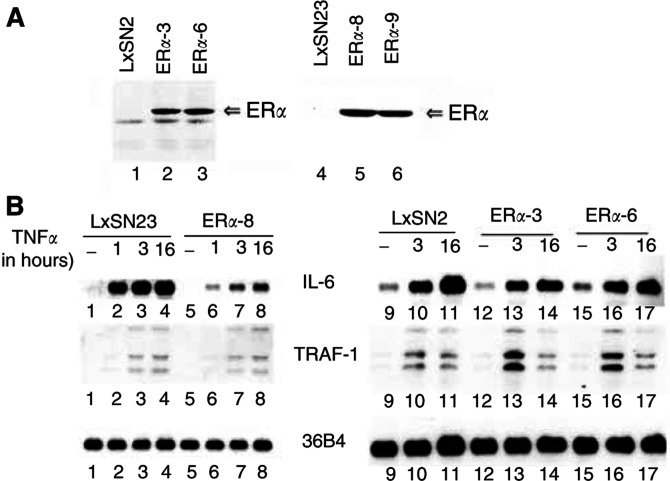
Oestrogen receptor alpha reduces TNF*α*-inducible IL-6 but not TRAF-1 expression in MDA-MB-231 cells. (**A**) Oestrogen receptor alpha expression in MDA-MB-231 cells. Oestrogen receptor alpha expression in cells transduced with retrovirus without the ER*α* coding sequence (LxSN2 and LxSN23) or with the ER*α* coding sequence (ER*α*-3, ER*α*-6, ER*α*-8 and ER*α*-9) was measured by Western blotting. Note that ER*α*-3 and ER*α*-6 cells express the wild-type receptor, whereas ER*α*-8 and ER*α*-9 cells express mutant receptor. (**B**) TNF*α*-inducible IL-6 but not TRAF-1 expression is lower in ER*α*-overexpressing cells compared to control cells. Cells were treated with TNF*α* for the indicated times and IL-6 or TRAF-1 expression was measured by Northern blot analysis. The same blot was reprobed with ribosomal protein gene 36B4 to ensure equal loading.

Previously, we reported constitutive NF-*κ*B activation in the ER*α*-negative breast cancer cell line MDA-MB-231, which correlated with increased expression of several NF-*κ*B-inducible genes including IL-6, Mn-SOD, cIAP-2 and TRAF-1 (Patel *et al*, 2000). In addition, using transient transfection assays, we showed transrepression of NF-*κ*B activity by ER*α* in these cells (Nakshatri *et al*, 1997). To further characterise the transrepression function of ER*α*, we stably overexpressed ER*α* in MDA-MB-231 cells using retrovirus-mediated gene transfer (Figure 1A

). Oestrogen receptor alpha-3 and ER*α*-6 clones overexpress wild-type ER*α*, whereas ER*α*-8 and ER*α*-9 overexpress mutant ER*α* (C530R), which cannot activate transcription of an ERE-containing reporter gene (data not shown). Cysteine 530 is within the recently identified KCK motif involved in intramolecular AF-1 and AF-2 interaction and this mutation reduces the affinity of ER*α* to oestrogen (E2) (Metivier *et al*, 2002). Cells expressing the mutant protein were used to evaluate transrepression independent of coactivator competition and by a mutant ER*α* with reduced affinity to E2. We compared the TNF*α*-inducible expression of NF-*κ*B-regulated genes IL-6 and TRAF-1 in parental and ER*α-*overexpressing cells. Inducible IL-6 but not TRAF-1 expression was lower in ER*α*-3 and ER*α*-6 cells compared to LxSN2 cells (Figure 1B). Similarly, IL-6 expression was lower in ER*α*-8 cells compared to LxSN23 cells (Figure 1B). Tumour necrosis factor alpha (TNF*α*)-inducible expression of cIAP-2, another NF-*κ*B regulated gene, was not influenced by ER*α* suggesting that transrepression is promoter-context dependent (data not shown). Transrepression of Mn-SOD expression by ER*α* was observed with early passage cells but not in late passage cells, which suggests existence of inherent mechanism to overcome transrepression (data not shown). As mutant ER*α*(C530R) transrepressed IL-6 expression, sequestration of common limiting coactivators by ligand-activated ER*α* is not necessary for transrepression.

### Pure antioestrogen ICI182780 reverses transrepression function of ER*α*

**Figure 2:**
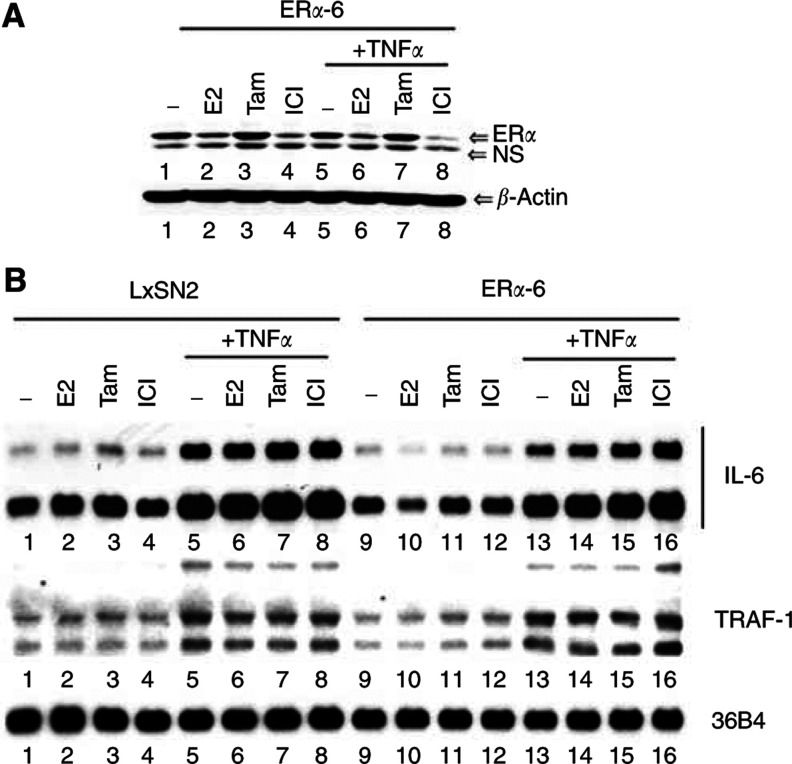
Pure antiestrogen ICI182780 overcomes the transrepression function of ER*α*. (**A**) Effect of oestrogen (E2), tamoxifen and ICI182780 on the stability of ER*α*. Oestrogen receptor alpha-6 cells were treated with E2 (10 nM), tamoxifen (1 *μ*M) or ICI182780 (100 nM) for 2 h followed by further incubation for 16 h with or without TNF*α*. Oestrogen receptor alpha protein level was determined by Western blotting. The same blot was reprobed for *β*-actin. (**B**) ICI182780 overcomes the transrepression function of ER*α*. RNA from cells treated as above was subjected to Northern analysis with IL-6 or TRAF-1 probe. Two exposures of the IL-6 blot are shown to highlight the effect of E2 on the basal IL-6 expression level in ER*α*-6 cells.

To further confirm the role of ER*α* in reducing TNF*α*-inducible expression of IL-6 in ER*α-*overexpressing cells, we preincubated cells with E2, tamoxifen or ICI182780 for 2 h and measured IL-6 expression with or without TNF*α* treatment for 16 h. Binding of E2 leads to activation and subsequent proteosome-dependent degradation of ER*α* (Lonard *et al*, 2000). Previous studies have shown that binding of tamoxifen to ER*α* leads to stabilisation, whereas binding to ICI182780 leads to degradation of ER*α* without activation (Ali *et al*, 1993). Tamoxifen stabilised, whereas E2 and ICI182780 reduced ER*α* level in ER*α*-6 cells (Figure 2A

). The effect of various ligands on basal and TNF*α*-inducible IL-6 and TRAF-1 expression was examined. Oestrogen reduced basal IL-6 expression in ER*α*-6 cells compared to LxSN2 cells (Figure 2B, middle panel). This could be due to sequestration and subsequent degradation of common coactivators by ER*α*. Interestingly, TNF*α*-inducible IL-6 expression was not influenced by E2. TNF*α*-induced IL-6 expression was still lower in tamoxifen-treated ER*α*-6 cells compared to tamoxifen-treated LxSN2 cells, suggesting that tamoxifen-bound ER*α* is capable of transrepression, consistent with our previous transient transfection studies (Nakshatri *et al*, 1997). In contrast, TNF*α*-inducible IL-6 expression was similar in ICI182780-treated ER*α*-6 and LxSN2 cells (Figure 2B, compare lanes 8 and 16). Note that none of the ligands altered TNF*α*-inducible TRAF-1 expression, which suggests that the observed effect of ligands on IL-6 expression is not due to toxicity. From these results, we conclude that ER*α* is responsible for lower IL-6 expression in ER*α*-6 cells compared to LxSN2 cells, and antioestrogens that destabilize ER*α* can overcome ER*α*-mediated suppression of IL-6 expression.

### TNF*α* reduces the stability of ER*α* protein

**Figure 3:**
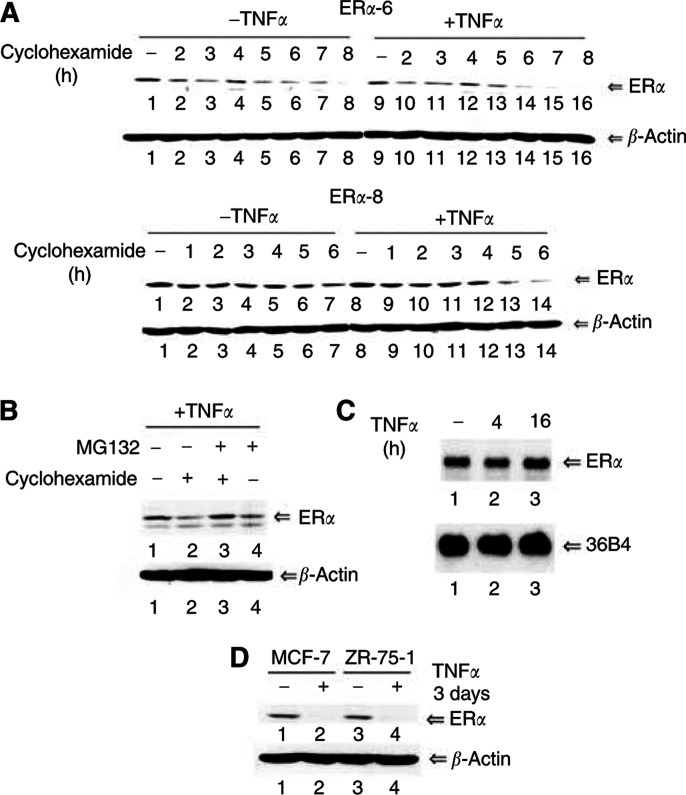
TNF*α* reduces stability of ER*α* protein. (**A**) Stability of ER*α* in untreated and TNF*α-*treated cells. Untreated or TNF*α*-treated (for 16 h) ER*α*-6 and ER*α*-8 were exposed to cyclohexamide (20 *μ*g ml^−1^), and cells were harvested at specific time intervals as indicated. Oestrogen receptor alpha protein was measured by Western blotting. Note reduced stability of ER*α* in TNF*α*-treated cells. (**B**) Oestrogen receptor alpha undergoes proteosome-mediated degradation in TNF*α*-treated cells. Oestrogen receptor alpha-6 cells were first treated with TNF*α* for 16 h. Subsequently, cells were incubated with cyclohexamide with or without MG132 for 4 h and ER*α* protein levels were measured by Western blotting. (**C**) Tumour necrosis factor alpha has no effect on ER*α* transcript levels. Oestrogen receptor alpha transcripts were measured by Northern blotting. (**D**) Tumour necrosis factor alpha reduces endogenous ER*α* in MCF-7 and ZR-75-1 cells. Oestrogen receptor alpha protein levels were measured after 3 days of TNF*α* treatment.

We consistently observed a lower level of ER*α* in TNF*α*-treated cells compared to untreated cells and a further enhancement of ICI182780-dependent degradation of ER*α* by TNF*α* (Figure 2A). In early passage cells, TNF*α* reduced ER*α* protein level by as much as 60% (data not shown). This raised the possibility that TNF*α* modulates transrepression function of ER*α* by inducing its degradation. Towards this end, ER*α*-6 and ER*α*-8 cells with or without prior treatment with TNF*α* for 16 h were incubated with cyclohexamide to block protein synthesis. Cells were harvested at specific time intervals and ER*α* protein was measured by Western blotting. Oestrogen receptor alpha stability was much lower in cells pretreated with TNF*α* compared to untreated cells (Figure 3A

). Oestrogen receptor alpha undergoes proteosome-mediated degradation in TNF*α*-treated cells as the proteosomal inhibitor MG132 prevented ER*α* degradation (Figure 3B). Neither caspase inhibitors nor calpain inhibitors altered the stability of ER*α* under untreated and TNF*α*-treated conditions (data not shown). Oestrogen receptor alpha transcript levels were similar in untreated and TNF*α-*treated cells, suggesting that the effect of TNF*α* on ER*α* is at the level of protein stability (Figure 3C). None of the effects of TNF*α* on ER*α* is due to TNF*α*-induced apoptosis of MDA-MB-231 as these cells were resistant to TNF*α* irrespective of ER*α* overexpression (data not shown). Tumour necrosis factor alpha-induced destabilisation of ER*α* was not restricted to MDA-MB-231 cells as TNF*α* reduced ER*α* protein in ER*α*-positive MCF-7 and ZR-75 cells (Figure 3D). Consequences of ER*α* degradation on transrepression in MCF-7 cells could not be studied because of lack of IL-6 expression in these cells and their sensitivity to TNF*α*-induced apoptosis (data not shown).

### Phosphatidylinositol-3-kinase inhibitor LY294002 stabilises ER*α* protein in MDA-MB-231 cells and inhibits TNF*α*-induced but not E2-induced degradation of ER*α* in MCF-7 cells

**Figure 4:**
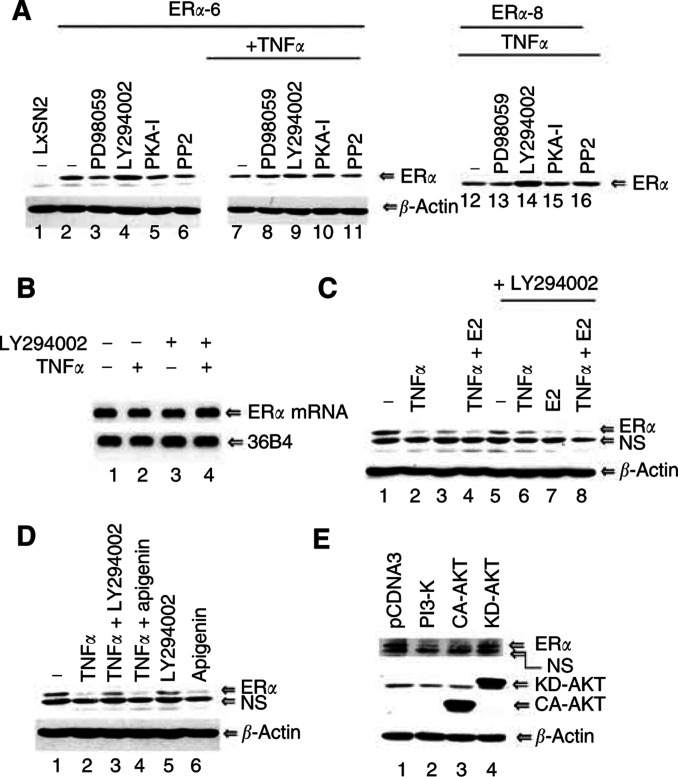
The PI3-kinase inhibitor LY294002 stabilises ER*α* protein. (**A**) The effect of MAP kinase, PI3-kinase, PKA and SRC kinase inhibitors on ER*α* stability. Oestrogen receptor alpha-6 cells were incubated with inhibitors of MAP kinase (PD98059, 20 *μ*M), PI3-kinase (LY294002, 20 *μ*M), PKA (2.5 *μ*M) or SRC (PP2, 5 *μ*M) with or without TNF*α* for 16 h. Oestrogen receptor alpha protein levels were measured by Western blotting. Similar results were obtained in TNF*α*-treated ER*α*-8 cells. (**B**) LY294002 has no effect on ER*α* transcripts as measured by Northern blot analysis. (**C**) LY294002 inhibits TNF*α*-induced but not E2-induced ER*α* degradation in MCF-7 cells. MCF-7 cells were treated with the indicated reagents for 3 days and ER*α* protein levels were measured as described above. NS=nonspecific. (**D**) Apigenin, a casein kinase II inhibitor, fails to inhibit TNF*α*-induced degradation of ER*α* in MCF-7 cells. (**E**) Constitutively active PI3-kinase and AKT (CA-AKT) but not kinase-dead AKT (KD-AKT) reduces the level of ER*α* in MCF-7 cells. Oestrogen receptor alpha levels in cells transfected with the indicated expression vectors using lipofectamine reagent for 48 h is shown.

MAPK, cyclin A/cdk2, AKT, RSK2, PKA, PAK1, p38 kinase and Src phosphorylate ER*α* (Ali *et al*, 1993; Lee and Bai, 2002; Wang *et al*, 2002). Phosphorylation leads to ligand-independent activation in most cases, and activated ER*α* undergoes coactivator-ubiquitin-dependent degradation (Lonard *et al*, 2000). Phophatidylinositol-3-kinase, which is upstream of AKT, as well as MAPK are constitutively active in MDA-MB-231 cells and may promote phosphorylation-dependent degradation of ER*α* (Ma *et al*, 2001; Sliva *et al*, 2002). Consistent with this possibility, ER*α* showed ligand-independent activity in ER*α*-3 and ER*α*-6 cells (data not shown). To investigate whether any of these kinases determine the stability of ER*α* and thus modulate transrepression function, we treated cells with various inhibitors for 16 h with or without TNF*α* treatment and measured ER*α* protein. The MAP kinase inhibitor PD98059, PKA inhibitory peptide or Src kinase inhibitor did not alter ER*α* protein level in ER*α*-6 cells (Figure 4A

). In contrast, the PI3-kinase inhibitor LY294002 stabilised ER*α* protein under both untreated and TNF*α*-treated conditions. Similar results were obtained in ER*α*-8 cells. Increase in ER*α* protein in LY294002-treated cells was not due to increased transcription of ER*α* in LY294002-treated cells (Figure 4B). To further confirm the role of PI3-kinase on ER*α* stability, we examined the effect of LY294002 on TNF*α*-induced degradation of ER*α* in MCF-7 cells. Although LY294002 reduced the basal ER*α* protein level possibly due to its effects on general transcription, it blocked TNF*α*-induced but not E2-induced degradation of ER*α* (Figure 4C). Recent studies have shown that LY294002 inhibits both PI3-kinase and casein kinase II (Davies *et al*, 2000). We used apigenin, a casein kinase II inhibitor (Critchfield *et al*, 1997), to support our conclusion that PI3-kinase is involved in TNF*α*-induced degradation of ER*α*. Apigenin failed to inhibit TNF*α*-induced degradation of ER*α* in MCF-7 cells (Figure 4D). In fact, apigenin on its own reduced ER*α* level. To further confirm the role of PI3-kinase in destabilisation of ER*α*, we transfected MCF-7 cells with constitutively active PI3-kinase and measured ER*α* protein 48 h after transfection. Oestrogen receptor alpha protein levels were lower in cells transfected with PI3-kinase expression vector (Figure 4E). Similar results were obtained in 293 and MDA-MB-231 cells transfected with ER*α* and constitutively active PI3-kinase (data not shown). Activation of AKT alone is sufficient for PI3-kinase-mediated destabilisation of ER*α* as a constitutively active AKT (CA-AKT) but not kinase-dead AKT (KD-AKT) reduced ER*α* levels (Figure 4E). It is possible that PI3-kinase-mediated destabilisation of ER*α* involves AKT-dependent phosphorylation of ER*α* followed by activation-coupled degradation. Our repeated attempts to establish MDA-MB-231 cells overexpressing ER*α* mutants that cannot be phosphorylated by cyclin A/cdk2 (S102N,104P,106A), MAPK/cdk7 (S118A), AKT/RSK (S167A), PKA (S236A) or SRC (Y537F) were not successful. In transient transfection assays, phosphorylation-defective mutants were always expressed at a higher level than wild-type ER*α* (although expressed from a same promoter), suggesting that phosphorylation-defective mutants are more stable than wild-type ER*α* (data not shown).

### Prolonged exposure of ER*α*-overexpressing cells to TNF*α* leads to loss of transrepression, which can be reversed partially by LY294002

**Figure 5:**
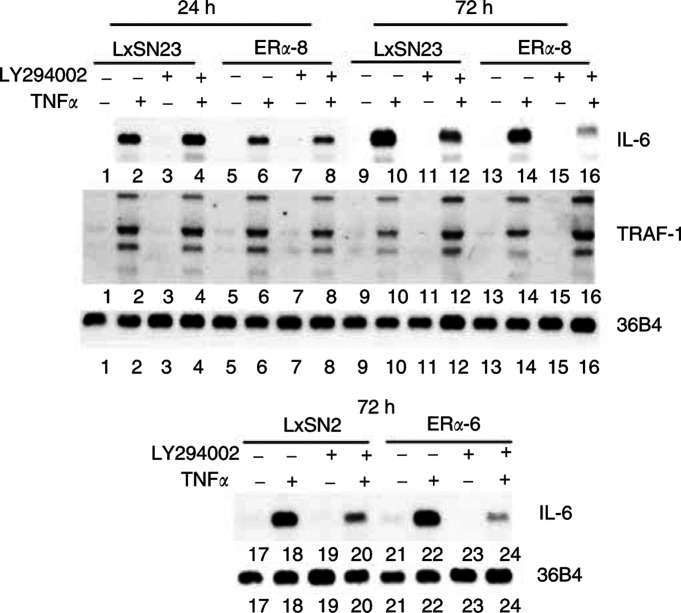
LY294002 inhibits TNF*α*-induced IL-6 but not TRAF-1 expression. Cells were treated with TNF*α* for 16 h or 3 days with or without LY294002. The media was changed daily with the addition of fresh TNF*α* and LY294002. Interleukin-6 and TRAF-1 expression levels were measured by Northern analysis.

To determine the consequences of stabilisation of ER*α* by LY294002 on transrepression, we treated parental and ER*α*-overexpressing cells with TNF*α* for 16 h or 3 days and measured IL-6 expression levels. Interleukin-6 expression in ER*α*-8 cells was lower than in parental cells after 16 h of TNF*α* treatment (Figure 5

). However, IL-6 expression was similar in both LxSN23 and ER*α*-8 cells after 3 days of TNF*α* treatment. Interestingly, LY294002 was more effective in reducing TNF*α*-inducible IL-6 expression in ER*α*-8 cells compared to LxSN23 cells. Similar results were obtained in ER*α*-6 cells. Note that LY294002 had no effect on TNF*α*-inducible expression of TRAF-1. Thus, inhibition of TNF*α*-inducible IL-6 expression by LY294002 is less likely due to reduction in AKT/PKB-mediated activation of NF-*κ*B or toxicity. We propose that LY294002 reduces TNF*α*-inducible IL-6 expression in ER*α*-overexpressing cells by enhancing transrepression function of ER*α*. In contrast to enhanced transrepression, LY294002 reduced transactivation by ER*α* (data not shown, Kishimoto and Nakshatri, submitted). Surprising specificity of LY294002 in inhibiting IL-6 but not TRAF-1 expression is encouraging as inhibitors with similar properties can be used to reduce invasion of breast cancer cells, more so of ER*α*-positive cancer cells, by specifically reducing IL-6 expression.

## DISCUSSION

In this report, we show that the ER*α* protein level in breast cancer cells is regulated by TNF*α* and PI3-kinase, which has important implications on the transrepression function of ER*α*. Transrepression by ER*α* is believed to be responsible for reducing invasion and metastasis of ER*α*-positive breast cancers (Platet *et al*, 2000). Repression of gene expression appears to be a major function of ER*α* as recent studies show that among ∼400 genes regulated by ER*α* in MCF-7 cells, ∼70% of them are downregulated (Frasor *et al*, 2003). By lowering the ER*α* protein level, TNF*α* and PI3-kinase can overcome transrepression by ER*α*, thus promoting invasion and metastasis of breast cancers. Recent molecular profiling data with patient samples is consistent with the above observation. Patients with lower levels of ER*α* protein in their tumours have shorter disease-free survival rates than patients with higher levels of ER*α* in their tumours (Sorlie *et al*, 2001). It is interesting that PI3-kinase levels are higher in highly invasive and metastatic breast cancer cell line MDA-MB-231 cells compared to nonmetastatic MCF-7 cells (Sliva *et al*, 2002), which can explain for LY294002-induced stabilisation of ER*α* in MDA-MB-231 cells overexpressing ER*α*. Although our efforts to generate MCF-7 and MDA-MB-231 cells lacking PI3-kinase using siRNA technique were not successful, the failure of other kinase inhibitors including PD98059, PP2 and apigenin to alter the stability of ER*α* suggests that specific inhibition of PI3-kinase is responsible for LY294002-induced stabilisation of ER*α*. The PI3-kinase pathway appears to be involved in destabilisation of ER*α* by TNF*α* but not E2, as LY294002 inhibited TNF*α*-induced but not E2-induced degradation of ER*α* in MCF-7 cells (Figure 4). Our results differ to some extent from a recent report, which showed enhanced turnover of unliganded and liganded ER*α* in MCF-7 cells treated overnight with LY294002 (Marsaud *et al*, 2003). Authors suggested that PI3-kinase activity is required for stabilisation of ER*α* in MCF-7 cells. Some of the effect of LY294002 on ER*α* protein level in MCF-7 cells could be at the level of ER*α* transcription from the endogenous promoter as we also observed similar decrease in ER*α* protein in MCF-7 cells treated with LY294002. However, this is not the case when ER*α* is expressed through a heterologous promoter as LY294002 stabilised ER*α* in MDA-MB-231 derivatives ER*α*-6 and ER*α*-8 cells (Figure 4A). Several recent studies suggest that data generated using LY294002 alone should be interpreted cautiously. For example, LY294002 inhibits both PI3-kinase and casein kinase II at same concentration (Davies *et al*, 2000). LY294002 has been shown to bind to the LBD of ER*α* and inhibit its activity (Pasapera Limon *et al*, 2003). To ensure a direct role for PI3-kinase in destabilisation of ER*α*, we determined the effect of overexpression of constitutively active PI3-kinase on ER*α* protein levels in MCF-7 cells. PI3-kinase reduced ER*α* protein level in these cells (Figure 4E).

A major implication of this study is on sensitivity of breast cancer cells to chemotherapy. Interleukin-6 has been shown to increase motility and confer multidrug resistance to breast cancer cells (Tamm *et al*, 1991; Conze *et al*, 2001). By reducing basal and/or TNF*α*-induced IL-6 expression, ER*α* can reduce multidrug-resistant growth of breast cancer cells. Consistent with this possibility, in preliminary studies, we have observed increased sensitivity of ER*α*-overexpressing MDA-MB-231 cells to doxorubicin (data not shown). PI3-kinase inhibitors may further enhance the sensitivity of ER*α*-expressing cells to chemotherapy by stabilising ER*α*. We have recently observed inhibition of the transactivation function of ER*α* in tamoxifen-resistant breast cancer cells by PI3-kinase inhibitors (Kishimoto and Nakshatri, submitted). From these results, we propose that PI3-kinase inhibitors have the potential to overcome the multidrug resistance of ER*α*-positive breast cancers by simultaneously increasing transrepression and reducing transactivation by ER*α*.

How TNF*α* and PI3-kinase promote degradation of ER*α* remains to be determined. Although TNF-induced degradation of ER*α* has been reported, to our knowledge, this is the first report demonstrating a role for PI3-kinase in ER*α* degradation (Danforth and Sgagias, 1993). Phosphatidylinositol-3-kinase has recently been shown to be required for degradation of *β*-arrestin-1 in response to chronic insulin treatment (Dalle *et al*, 2002). It is possible that TNF*α* and PI3-kinase induces the expression of a protein that targets ER*α* for proteosome-mediated degradation or it may induce the activity of proteosomal subunits, which alters the specificity of the proteosome. In this regard, it has been shown that TNF*α* increases ubiquitin-conjugating activity by increasing the expression of UbcH2 through NF-*κ*B (Li *et al*, 2003). The other possibility is that TNF*α* alters the NEDD8 pathway, which has recently been shown to be involved in ER*α* degradation (Fan *et al*, 2003). Peroxisome proliferator-activated receptor gamma (PPAR*γ*) and aryl hydrocarbon receptor also promote proteosomal degradation of ER*α* (Qin *et al*, 2003; Wormke *et al*, 2003). It is possible that TNF*α* utilises these receptors to promote ER*α* degradation. In this regard, kinetics and degree of TNF and aryl hydrocarbon receptor-induced degradation of ER*α* are similar. The other possibility is that TNF*α*, like IFN*α*, induces the replacement of proteosomal subunits, resulting in altered proteolytic specificity (Hisamatsu *et al*, 1996).

Although interaction between NF-*κ*B and ER*α* was reported about 8 years ago (Stein and Yang, 1995), how that interaction leads to either transactivation or transrepression is not known. Initial studies suggested that competition for limiting coactivators is responsible for transrepression. However, subsequent studies by Parker's group and this study with a mutant ER*α* rule out coactivator competition as being the primary mechanism of transrepression, at least in breast cancer cells (Sheppard *et al*, 1999; Harnish *et al*, 2000; Valentine *et al*, 2000). Also, our studies show that not all NF-*κ*B-regulated genes are transrepressed by ER*α*, which suggests that transrepression involves specific promoter context. There may be similarity in transrepression by GR and ER*α*. Expression of IL-8 upon TNF*α* stimulation involves NF-*κ*B-dependent assembly of the transcription preinitiation complex followed by phosphorylation of the RNA polymerase II carboxyl terminal domain. Glucocorticoid receptor has been shown to interfere with phosphorylation of the RNA polymerase II carboxyl terminal domain without interfering with the preinitiation complex formation (Nissen and Yamamoto, 2000). Transrepression by GR in some instances involves corecruitment of the coactivator molecule GRIP1, and the coactivators SRC-1 and SRC-3 cannot substitute this function of GRIP1 (Rogatsky *et al*, 2002). Promoter specificity in ER*α*-mediated transrepression may also involve a similar mechanism.
